# Experiences of Using an Electronic Health Tool Among Health Care Professionals Involved in Chronic Obstructive Pulmonary Disease Management: Qualitative Analysis

**DOI:** 10.2196/43269

**Published:** 2023-03-30

**Authors:** André Nyberg, Anna Sondell, Sara Lundell, Sarah Marklund, Malin Tistad, Karin Wadell

**Affiliations:** 1 Department of Community Medicine and Rehabilitation Section of Physiotherapy Umeå University Umeå Sweden; 2 School of Education, Health and Social Studies Dalarna University Falun Sweden

**Keywords:** COPD, eHealth, internet, web based, health care professionals, primary care, pulmonary, management, tools, chronic, clinical, support, care, electronic, implementation

## Abstract

**Background:**

Chronic obstructive pulmonary disease (COPD) is one of the most common and deadliest chronic diseases of the 21st century. eHealth tools are seen as a promising way of supporting health care professionals in providing evidence-based COPD care, for example, by reinforcing information and interventions provided to the patients and providing easier access and support to the health care professional themselves. Still, knowledge is scarce on the experience of using eHealth tools from the perspective of the health care professional involved in COPD management.

**Objective:**

The study explored the experiences of using an eHealth tool among health care professionals that worked with patients with COPD in their daily clinical practice.

**Methods:**

This exploratory qualitative study is part of a process evaluation in a parallel group, controlled, pragmatic pilot trial. Semistructured interviews were performed with 10 health care professionals 3 and 12 months after getting access to an eHealth tool, the COPD Web. The COPD Web, developed using cocreation, is an interactive web-based platform that aims to help health care professionals provide health-promoting strategies. Data from the interviews were analyzed using qualitative content analysis with an inductive approach.

**Results:**

The main results reflected health care professionals’ experiences in 3 categories: receiving competence support and adjusting practice, improving quality of care, and efforts required for implementation. These categories highlighted that using an eHealth tool such as the COPD Web was experienced to provide knowledge support for health care professionals that led to adaptation and facilitation of working procedures and person-centered care. Taken together, these changes were perceived to improve the quality of care through enhanced patient contact and encouragement of interprofessional collaboration. In addition, health care professionals expressed that patients using the COPD Web were better equipped to tackle their disease and adhered better to provided treatment, increasing their self-management ability. However, structural and external barriers bar the successful implementation of an eHealth tool in daily praxis.

**Conclusions:**

This study is among the first to explore experiences of using an eHealth tool among health care professionals involved in COPD management. Our novel findings highlight that using an eHealth tool such as the COPD Web may improve the quality of care for patients with COPD (eg, by providing knowledge support for health care professionals and adapting and facilitating working procedures). Our results also indicate that an eHealth tool fosters collaborative interactions between patients and health care professionals, which explains why eHealth is a valuable means of encouraging well-informed and autonomous patients. However, structural and external barriers requiring time, support, and education must be addressed to ensure that an eHealth tool can be successfully implemented in daily praxis.

**Trial Registration:**

ClinicalTrials.gov NCT02696187; https://clinicaltrials.gov/ct2/show/NCT02696187

## Introduction

Chronic obstructive pulmonary disease (COPD) is one of the most common chronic diseases of the 21st century and a leading cause of chronic morbidity worldwide [1]. COPD is typically treated and managed with pharmacological and nonpharmacological therapies in primary, secondary, or tertiary care [1-4]. Although nonpharmacological treatments, such as pulmonary rehabilitation and self-management interventions, are considered vital components of COPD management [2,5], we know today that several barriers exist that result in low access, uptake, and completion rates [6-8]. Therefore, overcoming these barriers and finding new and alternative strategies to facilitate evidence-based care in COPD management are highly warranted [9,10].

eHealth tools represent a promising way of delivering health services in COPD management [11-13]. The use of eHealth tools includes, but is not limited to, intervening, educating, and keeping track of a person’s health, resulting in several clinically relevant health benefits among patients with COPD [11,12,14-16]. For example, our group previously found that access to a web-based platform increased self-reported physical activity levels, COPD-specific knowledge, and altered disease management strategies among patients with COPD in primary care [17]. However, we also found that the use of the eHealth tool varied profoundly between patients, and the vast majority mainly used the platform at the beginning stages of their treatment [17]. Furthermore, motivation, comfort with information technology tools, and level of health literacy were identified as vital explanatory factors affecting usage of the eHealth tool over time [18], findings that are supported by a recent qualitative systematic review that determined the perception of eHealth among over 300 patients with COPD across 19 individual studies [19]. However, besides motivation and comfort with information technology tools, other factors, such as access to 1-to-1 contact with health care professionals, were also critical for encouraging use of eHealth tools among patients with COPD [19]. Regarding the latter, van Zelst et al [20] recently demonstrated up to a 3-fold increase in eHealth tool usage among patients with COPD if the tool was used together with health care professionals compared with those who used the eHealth tool independently. This indicates the vital role of the health care provider in supporting the use of eHealth tools among patients with COPD.

Importantly, these tools are also accessible and potentially relevant for users other than patients, such as health care professionals and informal caregivers [21,22]. Recently, eHealth tools have been put forward as a viable alternative supporting health care professionals in providing evidence-based care, for example, by reinforcing information and interventions provided to the patients and providing easier access and support to the health care professional themselves [23,24]. Furthermore, eHealth tools are considered a promising way for health care professionals to interact with and support patients and their families at a distance [25,26], and they may improve patient-related outcomes and health care utilization by providing self-management support for the patient and decision support for the health care professional [26]. Besides, attitudes toward using eHealth tools among health care professionals are generally positive. In a recent global survey among 1091 health care workers, 4 out of 5 health care professionals thought that using eHealth tools can reduce workload and save time for the clinician [27]. Yet, despite the potential benefits of eHealth tools for health care professionals, and the generally positive attitude toward using eHealth tools, knowledge is scarce on the practical experience of using eHealth tools from the perspective of the health care professional, specifically the health care professional involved in COPD management [24,28,29]. In addition, a need for further qualitative research was warranted in a recent meta-synthesis to understand the key ingredients that will facilitate a positive user experience of eHealth among health care professionals [29]. About the latter, designing eHealth tools using cocreation or participatory methods that engage the end users in the development and design of the eHealth tool is recommended but not often used within COPD research. Therefore, using an explorative qualitative design, this study explored the experiences of using an eHealth tool that was designed using a cocreative process [24] among health care professionals working with patients with COPD in a primary care setting.

## Methods

### Study Design

This exploratory qualitative study is part of a process evaluation in a parallel group (1:1 allocation) controlled pragmatic pilot trial [10,17], reported per the Consolidated Criteria for Reporting Qualitative Research (COREQ) guidelines [30]. The study was registered at ClinicalTrials.gov (NCT02696187).

### Ethical Considerations

Ethical approval was given by the Regional Ethical Board, Umeå University, Umeå, Sweden (Dnr: 2014-319-31, 2015-457-32). In addition, written informed consent was obtained from each health care professional before their enrollment in the study. Study data are not anonymous but were deidentified. No compensation was provided to study participants.

### Setting and Sample

A total of 10 health care professionals at 5 publicly funded primary health care centers (2 situated in northern Sweden and 3 in central Sweden) were invited to participate using convenience sampling. All 10 health care professionals accepted. Primary care was targeted, because it is where the vast majority of patients with COPD in Sweden are treated [31,32]. The senior manager at each primary care unit assisted in identifying health professionals eligible for participation in the study. Telephone calls or emails were used to approach potentially eligible health care professionals at the included centers to participate in the planned study. To qualify for inclusion, health care professionals, independent of profession (eg, nurses, physicians, physiotherapists, occupational therapists, or dietitians), should meet patients with COPD in their daily clinical practice and be willing to use an eHealth tool, the COPD Web, as part of their clinical praxis for at least three months. As part of the process evaluation, interviews were performed with health care professionals involved in COPD management at 3 and 12 months, the latter to capture the longitudinal long-term experience of using the eHealth tool.

### The eHealth Tool

The COPD Web is an interactive web-based platform that was cocreated with patients with COPD, their relatives, health care professionals, and researchers. The content of the COPD Web was in line with the nonpharmacological health promotion interventions recommended by the Swedish National Board of Health and Welfare’s national guidelines for COPD management [33]. The COPD Web consisted of 3 main sections, 1 directed at patients with COPD, 1 at their relatives, and another at health care professionals [24]. The COPD Web’s development and design and the experience and effects of using the COPD Web among patients with COPD have all been extensively described elsewhere [10,17,24].

**Figure 1:**
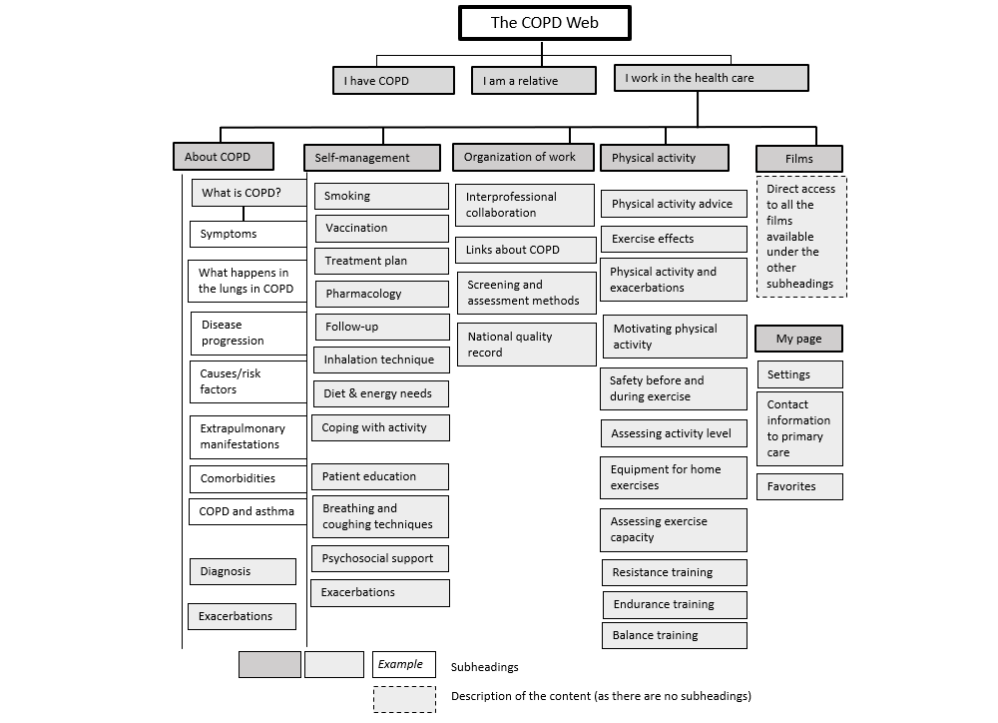
Overview of the content of the COPD Web sections for health care professionals. My page was a specific section of the COPD Web that became available when creating an account. In the My page section, the user could change settings, find contact information for their primary care center that was selected when creating the account, and find an overview of the “favorites” sections of the COPD Web. About the latter, each page of the COPD Web could be saved as a “favorite” and the user could have an overview of the sections that had been saved on the My page section. The purpose was to provide a quick and direct access to the specific content on the site being important for each user. COPD: chronic obstructive pulmonary disease.

The health care professional section of the COPD Web aims to support evidence-based care for patients with COPD, specifically self-management strategies. The section included factual texts, pictures, videos, and recommended and validated evaluation and screening tools [10]. An overview of the COPD Web’s current content, specifically, the section for health care professionals, is shown in Figure 1. Data on use of the COPD Web were gathered during the initial 3 months. Health care professionals made on average 15 (SD 19) log-ins to the COPD Web and spent 15 (SD 21) minutes on the site per log-in. Across the 10 health care professionals, the COPD Web was introduced to 102 patients with COPD during the 3 months.

### Introduction of the eHealth Tool

All health care professionals were given a 1-2-hour theoretical and practical face-to-face introduction to the COPD Web. The information provided was predetermined and similar across health care centers and independent of the profession of the health care professional. The introduction included information on the design, development, and purpose of the COPD Web. As part of the use of the COPD Web, the health care professionals were also instructed on how to introduce the COPD Web, using a prespecified routine (Textbox 1), to all patients with COPD that they met during the initial 3-month period. We went through all the steps highlighted in Textbox 1 with the health care professionals as part of the 1-2-h education, with the health care professionals also navigating the site. Besides the introduction of the COPD Web and in line with the pragmatic approach of the study, the health care professionals were free to use, or not use, or to adapt the use of the COPD Web as they deemed suitable or appropriate for each patient. No extra resources were provided to the primary care units, as health care professionals used the COPD Web as a part of their regular work practice [10].

**Routine introduction of the COPD Web to patients by health care professionals. Reproduced, with permission, from [10]. COPD: chronic obstructive pulmonary disease.**

- Registration and creation of an account to allow the patient to use the COPD Web.
- Introducing the website structure, the content of the main menus, and the functions of the website; for example, how to enlarge or reduce text, listen to the text, or bookmark information of particular interest.
- Introducing the “physical activity and exercise training” section to the patient. The health care professional will discuss the importance of physical activity/exercise training, point out the films with muscle strengthening exercises, and the page for registering physical activity (steps) with automated feedback.
- Introduction of 2 to 4 additional topics on the website of particular interest for the specific patient in question.
- Topics of specific interest for the patient will be noted on a leaflet with information about the COPD Web. The patient will receive the flyer and a card with the COPD Web’s URL address, username, and password.

### Research Team

The research group consisted of physiotherapists with different preunderstandings and insider and outsider perspectives of the eHealth tool. Two researchers, AN (PhD, male, 32 years) and MT (PhD, female, 43 years), conducted the interviews separately. Both interviewers were employed as postdoctoral researchers at the Department of Community Medicine and Rehabilitation, the section of Physiotherapy at Umeå University, Sweden, at the time of the study. Before the trial commenced, AN had conducted more than 20 interviews without specific prior training, while MT had conducted over 30 interviews under supervision during a previous postdoctoral employment. Before the interviews, there was no relationship between AN/MT and the health care professionals enrolled in the study. However, health care professionals knew that AN and MT had been involved in developing the COPD Web.

### Process of Data Generation

Semistructured individual interviews (except 1 made in pairs) with open-ended questions were conducted by AN or MT. Overall, 10 health care professionals (4 women), including 5 nurses, 2 physiotherapists, 1 dietician, 1 occupational therapist, and 1 physician, with a mean age of 50 (SD 11) years and 25 (SD 11) years of work experience, participated in the interviews; 5 health care professionals accepted interviews at 12 months. Reasons for declining an interview at 12 months included not using the eHealth tool (n=4) and being retired (n=1). Interviews were conducted in the health care professionals’ workplace at 3 months and over the telephone 12 months after receiving access to the COPD Web. In all interviews, only the interviewer and the health care professional were present. To ensure health care professionals’ privacy, all names were changed to pseudonyms during the start of the analysis so that only interviewers knew participants’ real names. No immediate callbacks on the interviews were conducted (ie, for potential amendments or additional questions). Still, exciting or unexpected topics raised during the interviews were discussed and used to guide follow-up questions during the following interviews.

The 3-month interviews ranged between 12 and 67 minutes (mean 39 minutes), while the 12-month interviews ranged between 10 and 29 minutes (mean 19 minutes). The interviews were structured by an interview guide (Multimedia Appendix 1), including questions about the professionals’ experiences using the eHealth tool, its applicability and usefulness, knowledge support and added value, and what they thought was missing from the eHealth tool when working with patients with COPD in primary care. For example, the first question was: “Tell me about if/how you have used the COPD Web (during this time)?” Participants were encouraged to speak freely in responding to the questions, and the interviews proceeded as conversations. Transcripts were not returned to health care professionals for comment or correction, and health care professionals were not engaged to provide feedback on the findings. All participants were assigned a pseudonym for transcription proofreading (used when quoting in the “Results” section).

### Data Analysis

Data from the interviews were analyzed using qualitative content analysis with an inductive approach, according to Graneheim et al [34,35]. Qualitative content analysis involves a stepwise, systematic analysis and a process of interpretation that focuses on similarities and differences found in the material, resulting in data organization into subcategories, categories, and potentially themes. This procedure is considered an appropriate method for illuminating health care professionals’ experiences of a complex phenomenon in a structured manner and is useful when dealing with already gathered qualitative data [34]. The unit of analysis was all interviews. One author (AS) who had not previously been engaged in the development of the eHealth tool or involved in data collection was chiefly responsible for data analysis. First, interviews were read through several times (with the assistance of audio recordings for auditory cues). Next, the transcripts’ content was divided into meaning units consisting of constellations of words and statements with the same meanings. Meaning units were then condensed and coded using Open Code software 4.03 [36] by one author (AS), with independently parallel coding conducted by 2 authors (KW and SM) in 2 interviews. Based on similarities and differences between codes, preliminary subcategories were clustered, abstracted, and merged into categories. The interpretive process was made in several steps and the analytical process involved a back-and-forth movement between the whole and parts of the texts. Through the analysis process, triangulation between researchers with different backgrounds was used to attain higher credibility [34]. All authors were involved in creating subcategories and categories, and changes were made until consensus was achieved. Trustworthiness was sought, for example, by all coauthors’ participation in several steps of the analysis, and the authors’ complementary competencies and perspectives were of great importance during analysis. In this study, the authors were all physiotherapists (AN, AS, MT, SL, SM, and KW) with clinical expertise in COPD (AS, SL, SM, and KW), specialist competence in COPD and exercise training (AN and KW), and scientific expertise in COPD (SL, KW, AN, SM, and MT), in eHealth (AN, SL, MT, SM, and KW), in exercise training/rehabilitation (AN, AS, MT, SM, and KW), and in qualitative research (AS, SL, MT, and SM). During discussions pertaining to data analysis, researchers critically reflected upon their prior understanding.

## Results

### Overview of Categories

**Figure 2:**
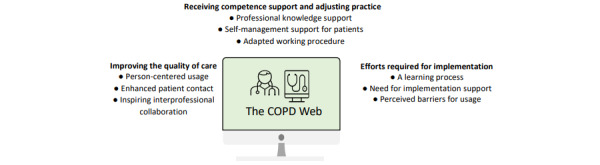
Overview of the main findings. Categories and subcategories.

The analysis resulted in 9 subcategories, grouped into 3 categories: receiving competence support and adjusting practice, improving the quality of care and efforts required for implementation, and representing the experiences of using an eHealth tool among health care professionals working with COPD (Figure 2).

### Receiving Competence Support and Adjusting Practice

#### Overview

The category addresses how the COPD Web provided competence support for health care professionals and patients, and how the work was adapted accordingly.

#### Professional Knowledge Support

The health care professionals communicated that the COPD Web was a complementing pedagogical and more extensive toolbox that could facilitate the patient meeting. They emphasized the COPD Web’s advantage as a concentrated, evidence-based, cutting-edge, and unified knowledge bank. Using the COPD Web led them to receive a higher and broader level of competence regarding COPD and provided support in patient education, which improved their ability to offer the patients more knowledge. In addition, the fact that the COPD Web provided patients with similar information as the health care professionals was perceived as an advantage.

The COPD Web was perceived to be a support when patients asked questions outside the health care professional’s specific area of expertise, which was considered reassuring. It was also considered more illustrative and spontaneous than using, for example, brochures in patient care.

It’s been a tool I’ve used spontaneously; instead of taking a textbook or chart or compendium, I've resorted to the COPD Web without really thinking about it.

Health care professional 1

Health care professionals experienced increased knowledge about physical activity and exercise, which facilitated prescription of exercise to patients. The health care professionals also expressed that they had gained increased knowledge about using scales and tests in assessing and evaluating aspects of patient health.

I knew nothing about them before, and I think that it’s been quite good to learn a bit about this Borg scale and how it can be used for cardio and strength training.

Health care professional 2

In addition to being a knowledge support in preparing for patient encounters, health care professionals suggested that the COPD Web would be specifically advantageous when introducing new personnel.

#### Self-management Support for Patients

Health care professionals emphasized that the COPD Web contained excellent self-management support for the patients (eg, practical tips for managing daily activities and efficient strategies for avoiding exacerbating symptoms). The health care professionals expressed that the patients who were more affected during their everyday lives were also more receptive to information and emphasized that well-informed patients with a higher level of knowledge about their disease also adhered better to the prescribed treatment. As a result of using the COPD Web, the health care professionals expressed that they now talked more about what the patient themselves can do in the event of deterioration and that they finally received positive responses regarding self-management from patients.

That’s where things changed! Goodness gracious. Because that's who I was on the phone with. That's where it clicked. How to take care of yourself, how to be active and the importance of both treatment and self-care and not to overexert yourself. Really, it’s a revolution. And it's actually fun.

Health care professional 1

According to the health care professionals, patients and relatives had shown great interest in the COPD Web, even though interest in the COPD Web was perceived to vary between patients. Younger patients and patients more affected by their disease in their everyday lives were perceived to be the most frequent users of the COPD Web. In addition, health care professionals described that patients now had more time to learn new and essential things concerning their disease thanks to the COPD Web, which could lead to more questions during appointments and better adherence to treatment. Furthermore, patients had expressed lessons they had learned using the COPD Web (eg, finally understanding their disease, feeling it is okay to live with COPD, and not having the same anxiety about it getting worse). Improvements in physical function and increased motivation by following their physical activity over time were also said by patients as positive consequences of using the COPD Web:

Don’t underestimate the part about competition and wanting to beat your own record and...even if you're a bit old and unwell and all, many people have a competitive streak and might think it's fun to compare and write up...but it can still be a bit of fun and...well, I think it can be fun.

Health care professional 3

Health care professionals emphasized that “patient stories” (the section of the COPD Web containing short video interviews with patients with COPD) were pedagogical in-patient education. Seeing other patients’ experiences and solutions provided valuable knowledge and support for patients:

If I’ve learned anything in my years in health care, I've seen that many times it can be a positive teaching method, I think, a patient telling something to another patient. So it's not just me, the health care professional, who is the storyteller.

Health care professional 4

#### Adapted Working Procedure

The COPD Web was perceived as logical, easy to navigate, and feasible to adapt to the patients’ needs. Further, it could help provide structure when meeting patients and include materials they could use in education classes of patients with COPD. Health care professionals expressed that they, in dialog, worked practically and reflected with the patient in connection with the COPD Web content. Specifically, videos were experienced as facilitating communication with patients and as making it easier for patients to absorb information.

Yes, the films are good because they come in several different ways, visual and auditory and maybe textual as well. They come to the patient via several channels, so to speak, making it very, very strong.

Health care professional 1

In addition, it was expressed that because of the COPD Web, finding suitable activities/exercises at the right level for different patients was easier. Assessments of physical function contained in the COPD Web also facilitated the prescription of exercises at appropriate levels. Furthermore, some health care professionals expressed that they had started to send out the standardized questionnaire with the usual invitation for the next checkup, as well as asking the patient to fill in “my COPD profile” in advance, leading to a more thorough consultation. As a result, health care professionals emphasized that they could meet the patient’s needs more directly and be better prepared than they had been before using the eHealth tool. Most health care professionals expressed that they would continue to use the COPD Web and work more with it in the future. One participant expressed that it was undesirable to work without the COPD Web:

Well, I couldn’t work without it. I just want to show it to everyone. So everyone can use it. All the doctors, everyone...so that they understand.

Health care professional 3

However, for some health care workers, the COPD Web did not affect the ways in which they worked or their dialog with patients. In addition, after the *12-month* intervention period, some health care professionals mentioned that they did not use the COPD Web as often and were not as structured as they had been during the initial months.

### Improving the Quality of Care

#### Overview

This category refers to the importance of enabling a person-centered usage or personalization of an eHealth tool, that is, the importance of the eHealth tool to enable an added value for the health care professional (of using the tool) which taken together can improve COPD management.

#### Person-centered Usage

Health care professionals emphasized the importance of a person-centered usage of the COPD Web. They described meeting patients at different stages of the disease, and the importance of being able to individualize the information given to the patient. They thought that the design of the COPD Web enabled this as the various sections of the COPD Web were applicable to patients of different ages and stages of the disease. However, individuals who considered themselves “healthy” indicated that they did not always recognize themselves in the information. Therefore, these health care professionals requested even more diverse information on the disease for different stages of disease severity. They pointed out that the information given to patients had to be individually tailored, dependent on a patient’s problems, needs, abilities, prerequisites, and resources. Furthermore, it was important that the information provided sounded familiar, and that patients could see themselves in what was presented.

I think...the important thing is that it resonates. Even if it’s not that particular activity, there’s something you can relate to. So I think it’s good and illustrative. Then you can fill it in yourself. You can’t have everything on film. Nah.

Health care professional 5

The health care professionals described that (when meeting a patient) the information on the COPD Web was chosen based on the patients’ individual needs and how they used the COPD Web was adapted to the patient in front of them. They further expressed that individually tailored (physical) meetings were still crucial when motivating patients to self-manage their health, as specific strategies are challenging to illustrate on video and in writing (eg, how to divide an activity into partial tasks for the purposes of energy conservation).

Fur future development, it was recommended that the COPD Web could be further developed regarding its content nutrition and emotional support.

Maybe you can clarify something on the front page...COPD is a varied disease and some need more help than others, and this page is adapted for the whole range, so some of what is written apply to those who are very sick.

Health care professional 6

#### Enhanced Patient Contact

The health care professionals described that using the COPD Web had initially resulted in more extended visits. However, they pointed out that conveying information must be allowed to take time—time that was considered well-invested, as they were confident that the extra time they spent using the COPD Web would pay off in the future through healthier patients.

It contains more information than what I’ve given before, so it’s a qualitatively better meeting than the 45 minutes I gave before. Now, I spend an hour. And I think...I’m quite sure I’ll it will pay of next time.

Health care professional 1

The health care professionals that used the COPD Web frequently described fewer emergency visits for the patients at the health care centers in which they worked, because they had changed their working procedures to be more aimed at prevention. In addition, the COPD Web facilitated telephone counseling and led to more telephone follow-ups instead of physical visits, which saved time. The health care professionals that used the COPD Web more frequently were convinced of its benefit and security for the patient. After using the COPD Web, some health care professionals described they wanted to reintroduce annual visits for patients that they had not hitherto prioritized, due to lack of time.

#### Inspiring Interprofessional Collaboration

Health care professionals emphasized that the COPD Web could contribute to collegial and interprofessional collaboration at the health care center, and within the county council, and that this collaboration would be important in the future. They expressed that they now made more contacts with other health care professions working with patients with COPD at their health care center. Different health care professionals used parts of the COPD Web differently and discussed different topics with the patients.

But one of its strengths is that it is so broad, there is so much variety. Different skills and different parts.

Health care professional 3

Furthermore, although interprofessional collaboration was considered crucial, the health care professionals pointed out that the content of the COPD Web highlighted physiotherapists’ vital role in COPD management specifically, and that physiotherapists needed to take more responsibility for COPD management in the future. In addition, it was addressed that the occupational therapist has a vital role regarding, for example, energy conservation techniques, and should therefore be involved more. They further expressed a wish to extend the content of the COPD Web to include hospital care, home care, and group treatments for COPD. Furthermore, health care professionals pointed out that some of the COPD Web’s information could be helpful to other patient groups, such as those with heart failure.

### Efforts Required for Implementation

#### Overview

This category presents the process of learning to use the COPD Web among health care professionals, what they found to be necessary for the tool’s implementation, and barriers they experienced when using the COPD Web.

#### A Learning Process

Health care professionals expressed that learning and becoming familiar with the COPD Web took some time, and thus, more time and information on how to use the tool initially was warranted. They especially needed to think through how to use the COPD Web and familiarize themselves with the information they wanted to show patients.

I’ve just browsed and looked around at what there is under different things, so that I can find it later.

Health care professional 3

It was further emphasized that it was important that the COPD Web operated smoothly and that it did not take too long to find what was needed when a patient was present in the examination room. Health care professionals pointed out that a delay when starting videos and too-long videos could lead to lost focus and disrupt the meeting with the patient. At *12 months*, the COPD Web seemed more integrated into daily work among those who continued to use the eHealth tool. Health care professionals became more accustomed to the tool the more often they used it during patient encounters.

#### Need for Implementation Support

Health care professionals expressed several matters they considered essential when implementing a tool like the COPD Web in daily clinical practice. First, they pointed out that changing routines and learning a new way of working take time. Thus, implementing new procedures in health care—specifically in primary health care with its complex and varied assignments—could be associated with resistance, especially when new methods initially take more time. Second, they further emphasized that health care professionals need to see that the tool is beneficial not only for the person with COPD but also for the health care professionals themselves during their everyday work.

You have to be sure that it benefits the staff. And if you're not sure, then you have a problem. I guess that applies to everything you introduce in terms of working methods.

Health care professional 1

Third, they emphasized that support from management is essential and the importance of proceeding cautiously in the event of novel work procedures. Besides, the significance of collegial support when introducing something new was emphasized. Fourth, a good introduction and education should not be neglected, including the opportunity to try out and use the tool in practice. Lastly, the health care professionals expressed the importance of using role models when implementing an eHealth tool such as the COPD Web (eg, intercollegial spread in health care centers where it is already being used and involving health care professionals who have already used it).

Perhaps spread it among those of us involved, in small groups to start with - don’t overreach but spread it small.

Health care professional 1

#### Perceived Barriers for Usage

Health care professionals perceived that a barrier to getting started with the COPD Web was when the work procedure was not anchored in primary health care centers in advance. In addition, they described not feeling involved in the decision to start using the COPD Web.

I think it was a bit crazy that we never talked about it here before, so it just sort of came and...nah, I don’t know. Feels like it’s still not really anchored in the health centre.

Health care professional 4

The health care professionals also felt the need for certain preconditions to be met before they could use the COPD Web in their clinical work (eg, a computer in the consulting room or that the patient was not too sick during the visit). A lack of time and needing to prioritize other tasks were also mentioned as obstacles to using the COPD Web. It was pointed out that if the COPD Web did not feel like a natural part of the patient visit, it was not used at all. The biggest obstacle to not using the COPD Web at the health care centers was patient related. The advantages of the patient using the tool at home were emphasized, but it was more difficult when they had no computer at home, or their computer skills were deemed too low. Computer skills were perceived to vary between patients, where greater computer skills seemed to be related not to gender, but to younger age and higher education. Although exceptions to this rule were sometimes apparent.

There’s...I met an 84-year-old man who showed me his computer driving licence, which he took 20 years ago, and he had no problems. And then there were sixty-year-olds who are not at all used to it.

Health care professional 2

## Discussion

### Principal Findings

This study explored the experiences of using an eHealth tool, the COPD Web, that was developed using a cocreative process among health care professionals working with COPD in primary care. The main results reflected study participants’ experiences in the following 3 categories: receiving competence support and adjusting practice, improving quality of care, and efforts required for implementation. The uniqueness of this study is the longitudinal design with both 3 and 12 months of follow-up, the use of an eHealth tool that was designed in a cocreative process, and the pragmatic trial design in which the qualitative analysis was part of a process evaluation. About the latter, the eHealth tool was used as part of daily clinical practice and except for a 1-2-h initial education no additional support was provided to the health care professionals, and they were free to use or not use the tool as they preferred. The findings of this study highlight that using an eHealth tool such as the COPD Web was experienced as providing knowledge support for health care professionals, leading to adaptation and facilitation of work procedures and person-centered care, enhanced patient contact, and encouragement of interprofessional collaboration. Taken together, use of the eHealth tool was experienced to improve the quality of care provided to the patient. Health care professionals also expressed that patients using the COPD Web were more well-informed and better equipped to manage their disease. The patients also adhered better to treatment, thus increasing their self-management ability. The latter is of utmost importance as self-management is one of the cornerstones of successful COPD management for which eHealth tools may play a vital part [2,16,18,26,37]. Furthermore, the 3- and 12-month data collection enabled novel insights into how the use of an eHealth tool could change over time, and that among those who continued to use the tool, it was now an integrated part of their daily clinical practice. Still, despite the positive experience of using an eHealth tool among health care professionals involved in COPD management, structural and external barriers requiring time, support, and education must be addressed to ensure that an eHealth tool can be successfully implemented in daily praxis.

### Interpretation of Findings

Numerous studies have investigated eHealth tools’ experiences among patients with COPD [18,38-40]. However, to our knowledge, this study is among only a few that has explored the experiences of using eHealth tools among health care professionals involved in COPD management [29,40]. Our results support previous research that claims that eHealth fosters collaborative interactions between patients and health care professionals, which explains why eHealth is a valuable means of encouraging well-informed and autonomous patients [41,42]. For example, in the category “improving the quality of care,” health care workers repeatedly expressed that using the COPD Web with their patients had resulted in better and more qualitative visits and more well-informed patients that were better equipped to handle their disease and adhered better to their treatment—increasing the patient’s ability to self-manage their disease. In addition, health care professionals emphasized the importance of “person-centered usage” of the COPD Web and the idea that the tool could be used to personalize treatment. For example, it was expressed that different sections and subsections of the COPD Web were necessary for patients at different ages and stages of the disease, and that the content could be adapted, and individualized, depending on which patient sat in front of them [43], thus highlighting the importance of individualization or “person-centered usage” of an eHealth tool as a potential key ingredient for a positive user experience among health care professionals [29]. Furthermore, similar to our findings, previous meta-analyses have demonstrated a positive relationship between an autonomy-supportive health care climate and the personalization of eHealth intervention contents, successful self-management, and behavior change [44,45].

Moreover, we have previously reported that a digital COPD education program could be used to increase objective measures of COPD-specific knowledge among health care professionals involved in COPD management [46]. Although objective measures of COPD-specific knowledge were not obtained in this qualitative study, in the category “receiving competence support,” health care professionals expressed that the COPD Web offered a unified knowledge bank, which led them to receive a higher and broader level of competence regarding COPD. Specifically, health care professionals expressed that their increased knowledge about physical activity and exercise facilitated the prescription of exercise to the patients, which is highly important considering the benefits of physical activity and exercise training in COPD [47-52]. Furthermore, inadequate professional competence, lack of person-centeredness, and limited access to evidence-based care have been identified as essential obstacles and barriers to prescribing exercise and physical activity interventions to patients [6,7,53,54]. Therefore, the notion that using an eHealth tool may increase competence, patient-centeredness, and facilitate exercise prescription is an important finding supporting the relevance of eHealth as a means of improving quality of care.

Health care professionals also perceived that the COPD Web contributed to collegial and interprofessional collaboration. For example, as expressed in the category “improving quality of care,” health care professionals now establish more contacts with other professionals working with patients with COPD at their health care center. Furthermore, considering the complexity of COPD management and the importance of an interdisciplinary treatment approach [2,55], if eHealth tools can facilitate interprofessional collaborations, eHealth could be used to tackle a key obstacle (low access to evidence-based care) to improving the quality of COPD services and care [56,57]. eHealth tools such as the COPD Web could be an essential means of reducing hierarchies, skepticism, and lack of knowledge about how other professions work and their roles in COPD management, both of which are known barriers to collegial and interprofessional collaboration [58,59].

Lastly, when implementing and using an eHealth tool for health care professionals as part of their clinical practice, our results align with previous research suggesting that there are likely to be initial barriers requiring time and education that need to be addressed [16,60-62]. For example, the category “efforts required for implementation” expressed a need for support and guidance, especially because additional time is necessary for learning to use the eHealth tool. Similar to our findings, a systematic review by Koivunen and Saranto [60] found that inadequate support and lack of training were critical obstacles to implementing and using eHealth tools among health care professionals. Comparable findings were expressed in a recent Cochrane qualitative evidence synthesis [61] on mobile eHealth tools, highlighting health care professionals’ need for education, support, and training when considering eHealth tools within clinical practice [60,61]. The need for initial training and education has also been reported among health care professionals involved in COPD management [62-64]; for example, Brewster et al [64] found that practical training and education were repeatedly seen as a facilitator for health care professionals to accepting and using eHealth tools and technologies.

Notably, the health care professionals in this study expressed that the biggest obstacle to using the eHealth tool was unrelated to the health care professionals themselves. Instead, it was related to whether patients did or did not have a computer at home or whether their computer skills were (perceived to be) too low. Several studies in the systematic review authored by Koivunen and Saranto [60] and other studies of various chronic diseases, including COPD [65-67], have highlighted that lack of access and skills are obstacles to using eHealth tools among patients. For example, we recently found that among patients with COPD enrolled in primary care, about 40% of eligible patients with COPD declined participation in an eHealth intervention due to no/limited experience with computers [17]. Furthermore, even among those accepting participation, and thus likely to consider themselves armed with sufficient technological skills, it was found that a higher need for technical support was identified as a primary barrier to usage among nonusers/seldom users of the eHealth tool during a 3-month intervention period [18]. Importantly, although it might be a primary barrier, we know from previous work on eHealth use among patients with COPD that continued use of eHealth tools among patients over time enables a transition from being insecure and experiencing technical concerns to acquiring technical confidence and improving disease management [68].

Nevertheless, it should be noted that the potential lack of access to computers and low computer skills among patients are not certain, as these were related via the experiences of health care professionals and were not expressed directly by patients. In a recent study, Sönnerfors et al [69] found that among patients with COPD in Sweden, over 90% had access to the internet, and 68% had access to a computer or laptop. Participants also had high knowledge of how to use the internet, with 91% having used the internet during the last 3 months and 85% almost every day. Taken together, indicating that although low access and computer skills are obstacles to using eHealth tools across various patient groups [65-67], it is vital that health care professionals do not draw firm conclusions based on their perception of the computer skills of their patients. Instead, a more relevant alternative for health care professionals would be to assess the level of health literacy among their patients to aid them in deciding whether the incorporation of an eHealth tool would be feasible. Health literacy has been identified as a vital explanatory factor affecting the usage of eHealth tools over time among patients with COPD.

### Strengths and Limitations

Methodological strengths in this study are its design following the COREQ guidelines, increasing the credibility of our findings [16]; that our interviewees were multidisciplinary regarding health care professions; and that no extra resources were provided to the primary care units as health care professionals used the COPD Web as a part of their regular work practice [10]. Furthermore, throughout the analysis process, triangulation between researchers with different backgrounds was used to achieve higher credibility [32], and all authors were involved in creating subcategories and categories, and changes were made until consensus was achieved. In addition, several strategies have been used to enhance trustworthiness [32,33]. First, interviews were conducted via a face-to-face meeting at the health care professional(s) workplace at 3 months and over the telephone at 12 months due to practical choices. The 2 interview methods are considered equally credible [47]. Interview times vary and, occasionally, are short. Still, we interpreted our data to be rich enough for the analysis performed here, and a specific duration is not a guarantee for richness [46,48]. Second, during the analysis, triangulation between authors was made to ensure that our interpretation was grounded in the empirical data [46]. In addition, we continuously consulted the audio recordings when triangulation indicated risks of interpretational differences in a transcript [29]. Lastly, even though the number of interviews is not a crucial criterion in qualitative methods, it should be noted that the number of health care professionals, especially at 12-month interviews, was small. Although the 12-month interviews were fewer and shorter, they were included in the analysis following the study protocol [10]. Notably, the 12-month interviews did enrichen the material, providing an important insight that among those who continued to use the COPD Web, the eHealth tool seemed more integrated into daily work than it had been after the initial 3-month period, aligning with previous research highlighting health care professionals’ need for time, as well as education, support, and training when implementing eHealth tools in clinical practice [60,61]. By contrast, we also found that although the eHealth tool was mainly considered positive during the initial 3-month follow-up, 4 out of 10 health care professionals did not use the tool at 12 months, thus indicating that additional strategies might be necessary to implement the tool in clinical practice successfully.

### Conclusions

This study is among the first to explore experiences of using an eHealth tool among health care professionals involved in COPD management. Our novel findings highlight that using an eHealth tool such as the COPD Web was experienced as providing knowledge support for health care professionals, leading to adaptation and facilitation of working procedures and person-centered care, enhanced patient contact, and encouragement of interprofessional collaboration—altogether improving quality of care. Furthermore, health care professionals emphasized that patients using the COPD Web were experienced to be better equipped to tackle their disease and adhere better to treatment—also increasing patients’ ability to self-manage their care. Lastly, before an eHealth tool can be successfully implemented within daily praxis, structural and external barriers requiring time, support, and education need to be addressed.

## Appendix

Multimedia Appendix 1 Guide to interviewing staff who have used the COPD Web. COPD: chronic obstructive pulmonary disease.

## Abbreviations

COPD: chronic obstructive pulmonary disease
COREQ: Consolidated Criteria for Reporting Qualitative Research

## Data Availability

The data sets generated and analyzed during this study are not publicly available to protect the participants’ confidentially. However, they are available from corresponding author upon reasonable request.
