# Supplementary material for: Experiences of Using an Electronic Health Tool Among Health Care Professionals Involved in Chronic Obstructive Pulmonary Disease Management: Qualitative Analysis
Source: JMIR Hum Factors. 2023 Mar 30;10:e43269. doi: 10.2196/43269 (PMC10131608; doi:10.2196/43269)
Supplement: Multimedia Appendix 1 [file humanfactors_v10i1e43269_app1.docx]

**Multimedia Appendix 1**

**Instructions for the interview guide**

The interview is part of the development/evaluation of the COPD Web. The purpose of the interview is to understand and gain knowledge about the experiences of healthcare professionals who have used the COPD Web. The guide is based on a narrative approach, i.e., the questions should be formulated so that they invite the research subject to tell freely about their experiences – if necessary, use exploratory follow-up questions such as "can you tell us more?" or "what did you think then?"

| **Interview parts** | **Examples of questions** |
| --- | --- |
| User habits (with starting question) | Tell me about if/how you have used the COPD Web (during this time)  For what purpose/purposes have you visited the COPD Web? |
| User experience | Have you identified yourself as a healthcare professional with the contents on the COPD Web?  How do you perceive the COPD Web to navigate/navigate on the Internet?  Have you missed anything in particular on the COPD Web?  How can the COPD Web be improved for you as a healthcare professional?  How have you perceived the [films/information/how information is presented/the amount of information]? |
| Potential contribution to clinical work | Have using the COPD Web had any impact on your interaction with the patient during consultation – both positive and negative?  Has using the COPD Web impacted your security in the professional role?  Has using the COPD Web impacted your collaboration with other professions? |
| Experiences of the eHealth tool when working with patients with COPD | How did patients experience using the COPD Web?  How did patients with COPD perceive the information on the COPD Web?  Was there any potential benefit of using the COPD Web among patients?  Could the content of the COPD Web be individualized to people with COPD with different disease severity/age/ gender/ degree of comorbidities etc.? |
| Future use | Is there anything you feel could be enhanced/developed on the COPD Web?  What are your thoughts about continuing to use the COPD Web in the future? |
| Usual finishing question | Is there anything else you have thought about/would like to say about the COPD Web that I have not asked about? |
